# The Level of Zinc, Copper and Antioxidant Status in the Blood Serum of Women with Hashimoto’s Thyroiditis

**DOI:** 10.3390/ijerph18157805

**Published:** 2021-07-23

**Authors:** Joanna Szczepanik, Tomasz Podgórski, Katarzyna Domaszewska

**Affiliations:** 1 Faculty of Health Sciences, School of Socio-Economics in Sroda Wielkopolska, 63-000 Sroda Wielkopolska, Poland; tomczewskaj@gmail.com; 2 Department of Physiology and Biochemistry, Poznan University of Physical Education, 61-871 Poznan, Poland; podgorski@awf.poznan.pl; 3 Faculty of Health Sciences, Calisia University, 62-800 Kalisz, Poland

**Keywords:** Hashimoto’s disease, oxidative stress, TBARS, zinc, copper

## Abstract

The aim of this study was to analyze selected indicators of oxidative stress. The study subjects consisted of 42 women with Hashimoto’s disease and a control group of 30 healthy women. The concentration of zinc (Zn) and copper (Cu) in the serum was determined by Atomic Absorption Spectrometry (AAS) and the total antioxidative potential by the Ferric Reducing Ability of Plasma (FRAP) method. In addition, an assessment of concentrations of thiobarbituric acid reactive substances (TBARS) and total phenolics was carried out. Our research showed a significant difference in TBARS concentration (*p* < 0.0001 (ES: 0.92)) without significant differences in Zn, Cu, FRAP and total phenolics concentrations. Analysis of the correlation of the obtained results of biochemical tests for both groups showed a highly significant dependence of FRAP and total phenolics concentration in the blood of the examined women (*r* = 0.5283, *p* = 0.0003). The obtained results indicate no differences in Cu, Zn, and FRAP concentrations in the blood between two analyzed groups and a significantly higher concentration of TBARS in Hashimoto’s thyroiditis women. The concentration of total phenolics significantly influences the value of the FRAP.

## 1. Introduction

Reactive oxygen and nitrogen species (RONS) are used by human organisms for such tasks as signaling, the neutralization of infectious microorganisms, the induction of apoptosis, the stimulation of antioxidants and repair processes [1]. Additionally, their excessive accumulation can impair the main molecules, including proteins, lipids and even nucleic acids, and inhibit their physiological function. In addition, oxidative damage incubated by RONS may play a role in thyroid disease [2]. There is a balance between the formation of RONS and their detoxification, known as redox homeostasis (intracellular reduction–oxidation). The most commonly generated ones are singlet oxygen, superoxide anion radical, hydroxyl radical, hydrogen peroxide, and nitric oxide [3]. Under physiological conditions, there are numerous enzymatic (superoxide dismutase (SOD), catalase (CAT), glutathione peroxidase (GPx) and glutathione reductase (GP)) and non-enzymatic (vitamin C, E, A, glutathione, bilirubin, uric acid, flavonoids, carotenoids, zinc, copper, and selenium) defense systems of the so-called antioxidants (present in erythrocytes of human blood serum, as well as in other biological fluids and tissues) aimed at preventing damage caused by RONS accumulation [3,4,5].

The inflammatory process within the thyroid, caused by Hashimoto’s disease, is at the same time the promoter of increasing the amount of RONS and causing disturbances in the oxidative–antioxidative balance [6]. Imbalance may increase inflammation and cell damage by stimulating the release of proinflammatory cytokines and changing enzyme functions [3,4]. Thyroid hormones play a key role in the regulation of oxidative metabolism. They increase mitochondrial respiration and produce free radicals. Furthermore, the presence of antioxidant defense system enzymes has been documented in the thyroid (such as SOD, GPX, CAT and non-enzymatic antioxidants) [7]. SOD catalyzes the conversion of superoxide molecules to H_2_O_2_ and oxygen. Depending on its form, it contains in the active center manganese (MnSOD) or copper together with zinc (Cu/Zn SOD) [8]. Thus, the availability of zinc and copper (SOD) may affect the body’s ability to defend against RONS [9]. As demonstrated in thyroid diseases, the level of these elements may be lowered in people with thyroid diseases [10,11]. The total antioxidant status (TAS)—defined as the total body’s ability to neutralize RONS—according to many authors, is lower in people with Hashimoto’s disease [12,13,14]. Polyphenols are other phytochemicals that provide protection against oxidative stress and free radicals [15]. Many authors suggest a significant correlation between elevated levels of compounds formed as a result of lipid peroxidation and thyroid diseases [16,17,18].

Substances reactive with thiobarbituric acid (TBARS) are formed as bioproducts of the oxidation of primarily polyunsaturated fatty acids, which are an integral component of biological membranes. Peroxidation reactions intensify during infection, inflammation, aging, and neurodegenerative and cancer diseases [19,20,21,22]. TBARS (including malondialdehyde (MDA)) can be an indicator of the body’s exposure to free radicals, and so a measure of oxidative stress.

This study is aimed at evaluating selected indicators of oxidative stress and the concentration of zinc and copper in women with Hashimoto’s thyroiditis in comparison to a healthy group.

## 2. Materials and Methods

### 2.1. Study Population

The study was conducted according to the Declaration of Helsinki and the National Statement and Human Research Ethics Guidelines and approved by IRB (Institute for Research in Biomedicine) at the Poznan University of Medical Sciences (10 May 2013; Ethics Approval Number: 302/13). All subjects gave their prior consent to take part in it. Participation in the research was voluntary, explained by full and reliable information about the nature, purpose and course of the study, as well as benefits and risks associated with participation.

Hashimoto’s disease is one of the most common causes of hypothyroidism, mainly among women aged 45–60 [23]. This is why the study subjects consisted of 42 women with Hashimoto’s disease (based on medical examinations, elevated antibody titers). The control group of 30 was selected from among healthy women (selected in terms of sex, age and body weight). Patient exclusion criteria (presence of at least one of the factors listed below): obesity, staying on a vegetarian or any other alternative diet, patients with active or post cancerous disease (ongoing radiation and/or chemotherapy treatment), patients with liver diseases (alanine transaminase (ALT) > 3x border line) except for patients with fatty liver disease, chronic kidney disease eGFR < 30 mL/1.73 m^2^/min, acute inflammation c-reactive protein (CRP) > 5 mg/dL, unstable ischemic heart disease, patients after an ischemic or hemorrhagic stroke (< 6 months), post STEMI (ST Elevation Myocardial Infarction) patients with a drug-eluting stent implantation, nSTEMI (No ST Elevation Myocardial Infarction) below 12 months, inherited metabolic disorders: phenylketonuria, galactosemia, autoimmune diseases (acute thyroiditis, celiac disease, systemic connective tissue disease, hemolytic anemia, vitiligo, Addison’s disease, hyperbilirubinemia), non-specific enteritis (Crohn’s disease, ulcerative colitis), pregnancy, psychological disorders, eating disorders such as anorexia and bulimia, antibiotic therapy, steroid therapy (ongoing), drug or alcohol addiction (a daily consumption of more than 1 portion of alcohol). All of the patients were asked not to take minerals (especially zinc and copper) or diet supplements, which could affect the measured biochemical blood parameters in the period before and during the study.

The baseline characteristics of both groups are shown in Table 1. Hashimoto’s disease was diagnosed based on value >35 U/L for anti-thyroid peroxidase (anti-TPO) antibodies and >20 U/L for thyroglobulin (anti-TG) antibodies [23,24]. In the group of women with Hashimoto’s disease, 45.2% managed to balance the level of hormones regulating thyroid function. A total of 76% of patients took synthetic thyroxine. After substitution treatment, the elevated level of thyroid-stimulating hormone (TSH) was still present in 11.9% of cases (normal range for TSH: 0.35–2.80 mIU/L), elevated anti-TPO titer in 50% and elevated anti-TG titer in 37.5%.

The average length of the illness is 7.7 years, and 19% are people diagnosed with the disease in less than one year.

The most frequently recorded health problems among women with Hashimoto’s disease are: weakness, fatigue, drowsiness, concentration disorders, mood swings, insomnia, lower libido, dry skin, headaches, problems with getting pregnant, intestinal problems.

### 2.2. Preparation of Blood Samples for Analysis

The subjects were asked to stop supplementation that could potentially affect the result. In the day before the examination, they were asked not to perform physical activity. Blood samples were taken from the ulnar vein using a S-Monovette syringe (Sarstedt, Nümbrecht, Germany), then placed in tubes containing a clot activator and centrifuged (1500× *g*, 4 °C, 4 min) to separate the serum (Universal 320R; Hettich Lab Technology, Tuttlingen, Germany). Samples were frozen and stored at −80 °C until analysis (U410, Ultra Low Temperature Freezer, New Brunswick Scientific, Enfield, CT, USA).

### 2.3. Determination of Copper and Zinc

The concentration of zinc and copper in the serum was determined by atomic absorption spectrometry (Atomic Absorption Spectrometry, AAS). 

For this purpose, a Perkin-Elmer, Model 3030 Zeeman flameless absorption spectrometer equipped with a HGA-600 graphite furnace and an AS-40 (Perkin-Elmer, Norwalk, CT, USA) automated sample dispenser was used.

Determination of copper and zinc was made in accordance with generally accepted standards: at the wavelength for zinc (213.9 nm), and for copper (324.8 nm). The accuracy of the method was determined in relation to material certified for Seron Trace Elements Serum (Nycomed Pharma As, Oslo, Norway) and for a measurement error of 3–5%. The measurement was repeated three times. The volume of the sample fed into the graphite cuvette of the sample was 20 μL. Reference values: zinc 70–120 μg/dL, copper 85–155 μg/dL [25].

### 2.4. Determination of Antioxidant Status

In order to determine the concentrations of total antioxidative capacity of plasma, the following methods were used: colorimetry (ability to reduce plasma iron concentration, FRAP, reference values: 600–1600 μmol/L) [26], concentration of reactive substances with thiobarbituric acid (TBARS, reference values: 1–6 μmol/L) [27] and total phenolic compounds (reference values: 2.8–4.0 (g GAE, gallic acid/L)) [28]. The samples were read using a multi-detection microplate reader (Synergy 2 SIAFRT BioTek, Winooski, VT, USA).

### 2.5. Statistical Analysis

All data are presented as median (interquartile range). Distribution was tested with the Shapiro–Wilk test for normality. Differences between variables were examined using the Mann–Whitney test. The relationship between the variables was tested while using Spearman’s rank correlation. The significance level for all statistical analysis was set at *p* ≤ 0.05. All results were statistically analyzed using Dell Inc. (2016), Dell Statistica v.13.—soft-ware.dell.com (Statistica 13, Statsoft, Dell, Tulsa, OK, USA). Effect sizes (ES) were calculated as the difference between means divided by the pooled standard deviation. Using Cohen’s (1988) criteria, an effect size ≥0.20 and <0.50 was considered small, ≥0.50 and <0.80 medium, and ≥0.80 large [29].

## 3. Results

Table 1 presents the anthropometric characteristics of the studied women, the average concentrations of the examined microelements and the concentration of indicators of reductive–oxidative status in women’s serum.

Comparative analysis of the level of biochemical markers determined in the blood at rest between two groups showed a significant difference in TBARS concentration (Mann–Whitney U-test, *p* < 0.0001, (ES: 0.92)), with no significant differences in Zn, Cu, FRAP and total phenolics concentrations. The TBARS level was significantly higher for women with Hashimoto’s disease (26.3 μmol/L compared to 3.2 μmol/L among healthy women). Analysis of the correlation of the obtained results of biochemical tests for both groups showed a highly significant dependence (positive correlation) of FRAP and total phenolics concentration in the blood of the examined persons (*r* = 0.5283, *p* = 0.0003). Additionally, a significant correlation was demonstrated between TBARS concentration and the presence of anti-TG in the studied group (*r* = 0.03312, *p* = 0.0321). There was no relationship between Zn and Cu concentration and oxidative stress indicators and serum anti-TPO and anti-TG levels.

## 4. Discussion

In our study, the most significant differences between people with Hashimoto’s disease and healthy people were related to the level of substances reactive with thiobarbituric acid, i.e., those resulting from lipid peroxidation (damage). We showed statistically significant differences in the TBARS level in both groups. For women with Hashimoto’s disease, the level of compounds formed as a result of lipid damage was significantly higher. Malondialdehyde (MDA) is the most significant among TBARS. In tissues, an increase in MDA concentration is observed depending on the increased production of RONS, and the resulting aldehyde has cytotoxic, mutagenic and carcinogenic effects [8]. Chakrabarti et al. also used MDA concentration assessment as a marker of oxidative stress [17]. These authors found that MDA levels were higher in patients with hypothyroidism prior to levothyroxine treatment and/or selenium supplementation than in the control group. MDA concentration has also been found to decrease after treatment and/or supplementation in patients with hypothyroidism. In addition, they obtained a significant positive correlation between the MDA level and baseline TSH values [17]. Other authors also obtained results indicating a higher level of MDA in patients with hypothyroidism [16,18].

In our study, serum zinc and copper levels were lower in women with Hashimoto’s disease compared to the control group, but the differences were not statistically significant. Borawska et al. suggest that in people with Hashimoto’s thyroiditis, the level of zinc in blood serum is reduced and it may be related to the ongoing inflammation of the thyroid gland and may result from insufficient intake of this element with the diet. In addition, they pointed out that the increase in anti-TPO titers was inversely correlated with the level of zinc in the blood serum of the studied women [30]. Therefore, with the decrease in serum zinc concentration, the titer of antithyroid antibodies increases, which may confirm the role of zinc in the functioning of the body’s immune defense [30,31]. Free triiodothyronine (FT3) and thyroxine (FT4) need zinc to fulfill their biological activity (as selenium and iodine), and a deficiency of this element negatively affects the metabolic activity of these hormones [32]. It is also possible that changes in the pool of stored elements, such as zinc, selenium and iodine, in the thyroid gland may affect the function of this gland depending on the secretion of TSH by the pituitary gland, responsible for the regulation of T3 and T4 hormones [33]. The studies showed that high levels of copper were associated with higher levels of both thyroid hormones—T3 and T4 [34]. Kucharzewski et al. proved that persons with thyroid disease (thyroid cancer, Graves-Basedow disease and nodular goiter) have significantly higher levels of copper in the blood compared to healthy people [35]. Sinha et al. obtained results that also indicate an elevated level of copper in people with hyperthyroidism [36]. Rasic-Milutinovic et al. showed that the concentration of copper in people with Hashimoto’s disease was significantly higher compared to healthy people. At the same time, they suggest that the ratio of copper and selenium may affect the level of thyroid hormones, and a higher selenium level and reduced copper may promote a reduction in L-thyroxine or cause euthyreosis at lower FT4 values [11]. Mittag et al. concluded that the association between copper and selenium in the blood serum may be a marker of thyroid hormone resistance (RTH) in adults [37]. Al-Juboori et al., however, did not find differences in the level of copper in the blood between patients with hypothyroidism and healthy people. They suggest that more research is required to determine whether the level of copper can affect the level of thyroid hormones [38,39].

At the same time, there is no coherence in the results of studies assessing the level of oxidative stress and the concentration of thyroid hormones in the blood [40]. Indicators of oxidative stress in hypothyroidism may be increased [41,42,43,44], reduced [45] or unchanged [46], whereas in subclinical hypothyroidism (defined as a high level of TSH at the normal values of FT3 and FT4, which is usually the initial stage of Hashimoto’s disease), the knowledge of oxidative stress is limited [2].

The FRAP assay includes many possible antioxidants present in blood: albumin, uric acid, bilirubin, vitamins C and E and phenolics. We observed correlation between FRAP and total phenolics for both groups. Reddy et al. found that FRAP was significantly reduced in patients with overt and subclinical hypothyroidism [47]. It was found that both hyperthyroidism and hypothyroidism are associated with increased oxidative stress, increased production of free radicals and oxidants has been shown [40,42,48,49,50], and at the same time, reduced resistance to oxidation has also been shown [42,48,51]. The most important effects of oxidative stress are: damage to mitochondria, a decrease in ATP (adenosine triphosphate) and glutathione, the breakdown of red blood cells, intracellular calcium homeostasis, the inactivation of some proteins, increased adenine nucleotide catabolism, an increase in the lipid peroxidation rate, depolarization, an increase in cell membrane permeability, DNA damage, the acceleration of cell apoptosis and changes in their functioning [8].

Recent research presented by Ruggeri et al. shows that in people with Hashimoto’s disease the biological antioxidant potential is lowered and the level of reactive oxygen metabolites is elevated compared to healthy people. They proved that the oxidative stress index (estimated as the ratio of the total amount of oxidants and antioxidants) in people with Hashimoto’s disease was statistically higher compared to the control group [12]. The results from 2019 confirm those obtained by the above-mentioned authors three years earlier [13], which suggests a clear imbalance between the endogenous production of free radicals and antioxidant defense, or the occurrence of oxidative stress in patients with thyroid disease, especially autoimmune inflammation of this gland [12]. An increased amount of RONS in the system of people with Hashimoto’s disease may be due to a decrease in the synthesis of enzymes with antioxidant activity (superoxide dismutase and glutathione), which is the result of lowering thyroid hormone levels. Furthermore, it is also known that hyperlipidemia, which develops with decreasing thyroid hormone levels, leads to an increase in RONS [14]. Total antioxidant status (TAS) and total oxidant status (TOS) reflect the general state of redox balance in the system [14]. Ates et al. compared the levels of TAS, TOS and the oxidative stress index (OSI) in the group of people with Hashimoto’s disease with overt and subclinical hypothyroidism, with euthyroidism and in healthy people. They obtained results in which TOS and OSI increased significantly in each phase of the disease and TAS decreased. In addition, there was a negative correlation between the level of antithyroid antibodies and the overall oxidative level [14,52,53]. The authors suggest that this is the first study that assesses oxidative stress at various levels of Hashimoto’s disease [14]. Similar results were obtained by other researchers. Baser et al. concluded that people with Hashimoto’s euthyroid disease had lower TAS and higher levels of TOS compared to healthy subjects, suggesting the role of oxidative stress in thyroid autoimmunity [51]. In the Wang et al. study, designed to determine the level of oxidative stress in patients with thyroid cancer, Graves’ disease, Hashimoto’s disease and the control group, TAS was lower, while TOS and OSI were higher in hypothyroidism than in a healthy control group [54]. Researchers suggest that the role of inflammation in oxidative stress can be explained in two ways: first, inflammation directly increases the level of hydrogen peroxide in thyroid epithelial cells, and second, it activates enzymes from the NADPH oxidase family in T and B lymphocytes that increase RONS production [54]. Another possible cause is that hypothyroidism is associated with a lower production of hormones secreted by this gland, and it is known that these hormones affect the synthesis and biological activity of antioxidative enzymes [55,56]. Increased levels of hormones as a result of a synthetic levothyroxine may reduce oxidative stress [55]. In a mentioned study conducted by Reddy et al., antioxidant defense in subclinical and overt hypothyroidism was evaluated and the reduction in antioxidant defense in overt hypothyroidism was found to be due to both reduced antioxidative enzyme synthesis and the low activity of these enzymes [47]. Thus, the possibility of preventing the development of overt hypothyroidism among people with Hashimoto’s disease by providing exogenous antioxidants should be considered. Additionally, future research projects in this direction should be carried out [14]. It is also required to investigate whether oxidative stress is the cause or the result of Hashimoto’s disease. Research results suggested that a lower value of TBARS is not dependent on a decrease in Zn and Cu values.

## 5. Conclusions

The results from this study indicate that:

1. There are no differences in the concentration of Cu and Zn in the blood of people with Hashimoto’s disease compared to the control group.
2. In the blood of patients with Hashimoto’s disease, a significantly higher TBARS concentration was found, with no difference in FRAP concentration.
3. The concentration of total phenolics has a significant positive effect on the value of the FRAP indicator.

## 6. Limitation of the Study

The study was performed on a small group of women. The study compared selected markers of oxidative stress in people with Hashimoto’s disease and healthy people; however, the relationship between the level of thyroid hormones and the assessed parameters was not analyzed in the study group. Future research is required to assess whether specific thyroid hormone values may modulate the level of the studied parameters and how this substitution affects treatment.

## Figures and Tables

**Table 1:** Anthropometric characteristics and comparison of zinc and copper levels and antioxidant potential in women’s serum.

Median (Interquartile Range).			
Parameters	Study Group (*n* = 42)	Control Group (*n* = 30)	*p* Value
**Age (years)**	40.0 ± 32.0–49.0	41.0 ± 34.0–35.0	0.3391
**Body weight (kg)**	61.0 ± 55.0–70.0	64.5 ± 58.0–72.0	0.1914
**Body height (cm)**	168.5 ± 163.0–172.0	168.0 ± 163.0–170.0	0.9905
**BMI (kg/m^2^)**	23.2 ± 19.3–24.7	22.1 ± 20.4–26.3	0.2881
**Zn (µg/dL)**	88.0 ± 82.0–95.0	86.5 ± 76.0–104.0	0.7217
**Cu (µg/dL)**	110.5 ± 100.0–122.0	110.0 ± 99.0–136.0	0.6908
**FRAP (μmol/L)**	694.1 ± 559.5–835.2	733.6 ± 617.4–855.4	0.4949
**TBARS (μmol/L)**	26.3 ± 20.1–35.6	3.2 ± 2.6–4.4	0.0000
**Total phenolics (g GAE/L)**	2.9 ± 2.6–3.2	3.1 ± 2.8–3.2	0.1857

Abbreviations: FRAP = Ferric Reducing Ability of Plasma, TBARS = thiobarbituric acid reactive substances, GAE = gallic acid.

## Data Availability

The data presented in this study are available on request from the corresponding author. The data are not publicly available due to the consent provided by participants on the use of confidential data.
